# Numerosity perception after size adaptation

**DOI:** 10.1038/srep32810

**Published:** 2016-09-21

**Authors:** Eckart Zimmermann, Gereon R. Fink

**Affiliations:** 1 Cognitive Neuroscience (INM3), Institute of Neuroscience and Medicine, Research Centre Juelich, Germany; 2 Department of Neurology, University Hospital Cologne, Germany

## Abstract

While some researchers propose the existence of a special numerosity sense, others challenge this view and argue that numerosity is derived from low-level features as density information. Here, we used size adaptation to manipulate the apparent area size of an object set without changing its physical density. After size adaptation, two probe patches were shown, each of which contained a specific numerosity of dots. Subjects were required to report, which probe patch contained more dots. Numerosity perception was compared between conditions where probe patches were adapted to appear smaller or larger. Size adaptation affected numerosity perception in a logarithmic fashion, increasing with the numerosity in the probe patch. No changes in density perception were found after size adaptation. Data suggest that size and density information play only a minor role in the estimation of low numerosities. In stark contrast, high numerosities strongly depend on size and density information. The data reinforce recent claims of separate mechanism for the perception of low and high numerosities.

Numerical cognition is a unique and universal neurobiological feature: Arithmetic competence seems to be even more basic than human language since it can be observed in infants as well as animals, which can discriminate the cardinality of sets^1^. Neural representations specifically dedicated to numerosity processing in the parietal^2^,^3^,^4^,^5^,^6^ and the prefrontal cortex^4^ have led to the proposal that numerosity perception may be a genuine sensory feature. The existence of a special numerosity sense has been suggested based on the finding that numerosity perception, as all other primary visual features, is susceptible to adaptation^7^. Inspecting for several seconds a patch containing a specific numerosity of dots leads observers to misperceive the numerosity of a subsequently shown patch of dots. Numerosity adaptation shares with many other adaptation effects a negative aftereffect: Adaptation to large numerosities decreases the perceived numerosity of the following patch, and vice versa.

The idea of a numerosity sense has been challenged, however, by others who argue that neurons may derive the numerosity of a set by visual cues, e.g. by calculating density and area^8^,^9^,^10^,^11^,^12^. In the first two studies the physical area of numerosity patches was manipulated resulting in concomitant changes of numerosity and density perception. The authors suggested that the change in global dot density, induced by varying area size, is responsible for the modulation of numerosity perception.

The aim of the present study was to investigate the role of area in visual numerosity judgments. In order to isolate perceived numerosity, density, and area, we here used visual size adaptation employing a method recently devised by Pooresmailie *et al*.^13^. Like other primary visual attributes, e.g., color, orientation, or motion, size is adaptable: The prolonged exposure to an object with a certain size changes the apparent size of a probe object subsequently shown. If size adaptation is applied before presenting a numerosity patch, the apparent size of the numerosity patch is manipulated while keeping physical stimulus attributes, i.e. dot density, constant.

We tested numerosity and density bias after size adaptation for a range from 4 up to 100 dots. It has been argued that the numerosity sense is limited to low numerosities, since once a certain threshold is reached, objects can no longer be resolved^14^. Consequently, previous studies suggest differential mechanisms for the perception of low and high numerosities. Estimation of a low (=32 dots) dot numerosity^15^ is independent of physical size mismatches, whereas estimation of a high (=128 dots) dot numerosity^8^ is not. Anobile *et al*.^14^ found that low but not high numerosity perception follows Weber’s law. They measured sensitivity for numerosity discrimination over a wide range of numerosities. Only for low but not for high numerosities, thresholds increased directly with numerosity, following Weber’s law. This range, in which numerosity discrimination followed Weber’s law reached up to 0.25 dots/deg^2^. The putative influence of size adaptation on numerosity judgments should thus increase with higher numerosities where the estimation relies more on visual cues than on a direct perception of numerosity.

## Results

**Figure 1:**
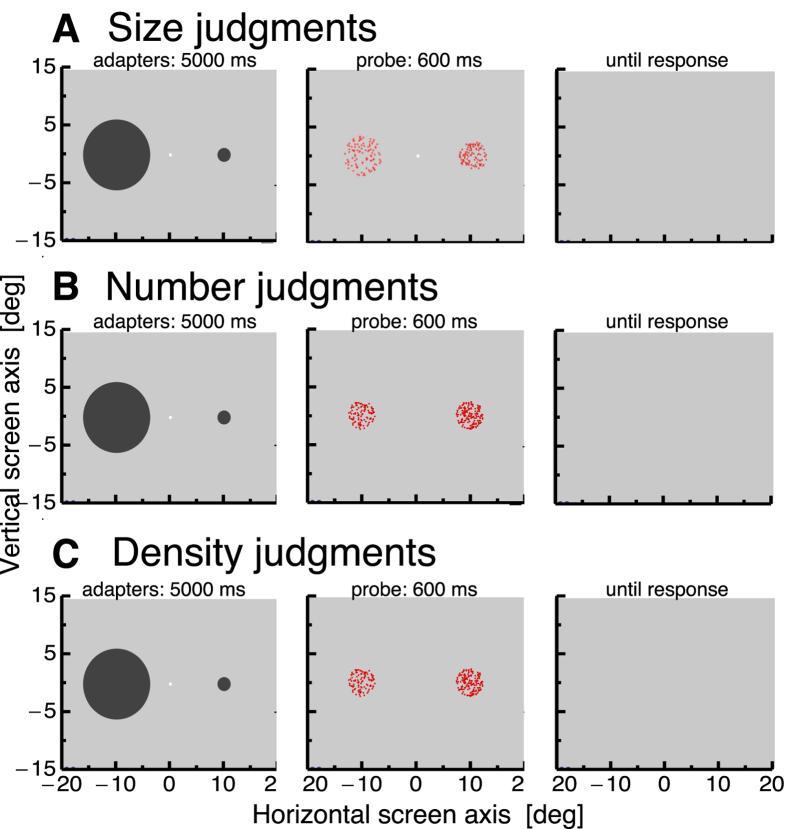
(**A**) Experimental set-up to test size judgments after adaptation to visual size. In the adaptation period two disks of different size were presented for 5000 ms. The disks changed contrast polarity to avoid the build-up of an afterimage. After adaptation two numerosity patches were presented for 600 ms. The patch on the right side was the probe and the other the reference. Subjects had to judge which patch appeared bigger. (**B**) Experimental set-up to test number judgments after adaptation to visual size. Identical procedure to (**A**), except that now the left patch varied in physical numerosity and subjects had to judge which patch appeared more numerous. (**C**) Experimental set-up to test density judgments a er adaptation to visual size. Identical procedure to (**B**), except that now subjects had to judge which patch appeared denser.

**Figure 2:**
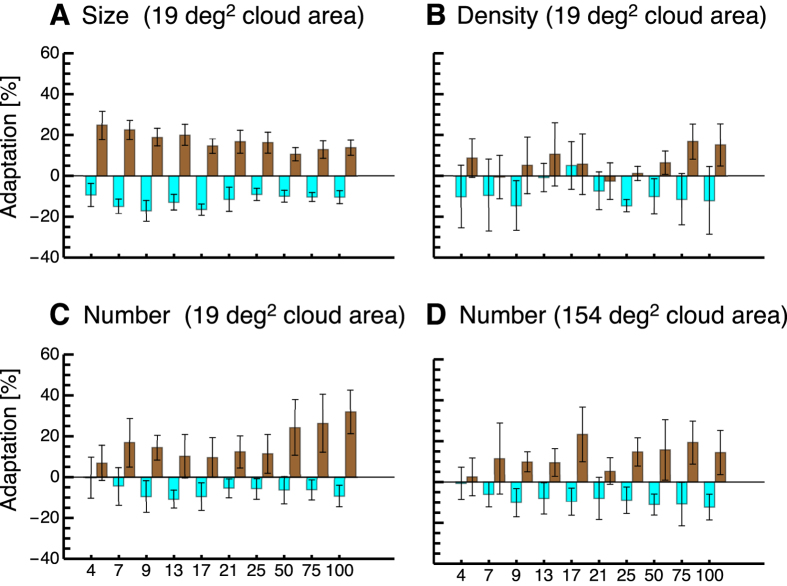
(**A–D**) Average biases in judgments of size, density and numerosity (for a 19 deg^2^ and a 154 deg^2^ probe cloud area) as a function of numerosity in the probe cloud. Data illustrated in brown color show biases after size adaptation with a small adapter disk and data illustrated in cyan color show biases after size adaptation with a large adapter disk. Positive values indicate overestimations and negative values underestimations. Error bars represent SEM.

We first tested whether size adaptation using disks as adapters can be induced for random dot probe patches (see Fig. 1A). The probe patch was always presented on the right side and the comparison patch on the left side. We compared apparent size of the probe patch between sessions in which the big adapter disk was on the left side and the small disk was on the right side, and vice versa. The big adapter disk should decrease the apparent size of the probe patch and the small adapter disk should increase it. For each tested numerosity in the probe patch (4–100) and for each of the three judgments (size, numerosity, density), we calculated a psychometric function. The point of subjective equality serves as an estimation of the bias. To quantify adaptation magnitude, we calculated the difference in bias for each subject from the session where the big adapter disk was on the right side and the session where the small adapter disk was on the right side. Size judgments after adaptation to a large adapter disk (shown in cyan) and numerosity judgments after adaptation to a small adapter disk (shown in brown) averaged across all subjects are illustrated in Fig. 2A. A 2 × 10 repeated measures ANOVA with the factors adaptation size (small/large) and numerosity (4–100) revealed a significant main effect for the factor adaptation size (F(1,5) = 132.58, p < 0.001) but no significant main effect for the factor numerosity (F(9,45) = 0.51, p = 0.722) and no interaction effect (F(9,45) = 1.34, p = 0.254). This result indicates that size adaptation with filled disks successfully modified the apparent size of random dot patches. However, there was no evidence that adaptation magnitude either depended on the numerosity in the probe patch.

We next wondered how numerosity judgments would be affected by size adaptation for the various numerosities in the probe patch (see Fig. 1B). We therefore asked subjects to estimate which of the two patches contained the higher numerosity of dots. Adaptation magnitude was calculated in the same way as for the size adaptation experiment. Numerosity judgments after adaptation to a large adapter disk (shown in cyan) and numerosity judgments after adaptation to a small adapter disk (shown in brown) averaged across all subjects are shown in Fig. 2B. Adaptation to size clearly changed numerosity judgments. However, this effect depended on the numerosity in the probe patch. Whereas low numerosities were mostly perceived veridically, estimations of high numerosities were strongly influenced by adaptation. Please see Demo 1 to have an impression of the effect. A 2 × 2 × 10 repeated measures ANOVA with the factors group (small number clouds/large number clouds), adaptation size (small/large) and numerosity (4–100) revealed a significant main effect for the factor adaptation size (F(1,9) = 43.24, p < 0.001) and a significant interaction effect between the factors adaptation size and numerosity (F(9,81) = 6.72, p < 0.001). The factor numerosity marginally failed to reach significance (F(9,81) = 1.84, p = 0.073). The significant interaction effect between the factors adaptation size and numerosity showed that adaptation magnitude indeed depended on the numerosity in the probe patch, such that higher numerosities were more strongly affected by adaptation. In order to test which numerosities were adapted significantly, we tested the difference between adaptation to small and large adapter patches against zero with paired t-tests, which were Bonferroni-corrected for multiple comparisons. We found significant adaptation (alpha-level = 0.005) for numerosities 13, 21, 50, 75, and 100. The p-values of these comparisons were: 4 dots, p = 0.291, 7 dots, p = 0.053, 9 dots, p = 0.059, 13 dots, p = 0.0019, 17 dots, p = 0.013, 21 dots, p = 0.002, 25 dots, p = 0.010, 50 dots, p = 0.0003, 75 dots, p = 0.001, 100 dots, p = 0.0001.

In separate sessions we also asked subjects to estimate which of the two random dot patches contained the higher dot density (see Fig. 1C). As can be seen in Fig. 2C, these data are all scattered around zero. A 2 × 10 repeated measures ANOVA with the factors adaptation size (small/large) and numerosity (4–100) revealed no significant main effect for the factor adaptation size (F(1,5) = 0.15, p = 0.716) nor for numerosity (F(9,45) = 1.29, p = 0.304) and no significant interaction.

**Figure 3:**
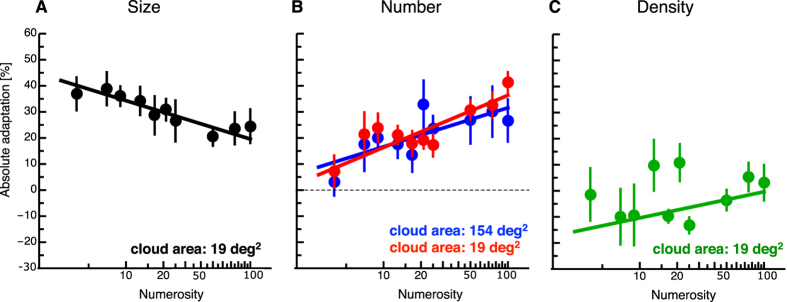
(**A–C**) Adaptation magnitudes for size (shown in black) numerosity of a small cloud area (shown in red), numerosity of a large cloud area (shown in blue) and density (shown in green) judgments after size adaptation as a function of numerosity in the probe patch. Data are fitted by logarithmic fit functions. Error bars represent SEM.

We then compared the absolute amount of size adaptation to the absolute numerosity and density adaptation magnitudes. This way, we aimed to investigate to what extent size adaptation predicted changes in numerosity judgments. In Fig. 3A we plot average absolute size adaptation (shown in black). Data are fitted with a logarithmic fit function (49.04–6.39* log(x), slope: p < 0.001). In Fig. 3B average absolute numerosity adaptation (shown in red for a small cloud area and in blue for a large cloud area). Data are fitted with a logarithmic fit function (small cloud area: −3.66 + 8.69* log(x), slope: p = 0.002, large cloud area: 1.70 + 6.49*log(x), slope: p = 0.02). Absolute adaptation is calculated for each subject by subtracting magnitudes after adaptation with the large adapter disk from magnitudes after adaptation with the small adapter disk and dividing the result by factor 2. Whereas size adaptation is strong also for small numerosities, numerosity adaptation gradually develops and is almost absent for the smallest numerosities. Interestingly, for high numerosities size adaptation of size judgments is even weaker than adaptation of numerosity judgments. Since in this analysis we were only interested whether size adaptation would have predicted the gradual increase in numerosity adaptation as a function of numerosity in the probe patch, we calculated the differences between absolute adaptation in size judgments and absolute adaptation in numerosity judgments for each subject. A oneway repeated measured ANOVA revealed a significant main effect of numerosity (F(9,45) = 4.433, p < 0.001). Post-Tests showed that the difference in adaptation of size and numerosity judgments for smaller numerosities was significantly higher than that for the higher numerosities. This analysis confirms that low numerosities are significantly less affected by size adaptation than higher numerosities. Figure 3C shows average absolute density adaptation. A logarithmic fit function was fitted to the data (20.42 + 4.35*log(x)), however no significant effect on slope was observed.

**Figure 4:**
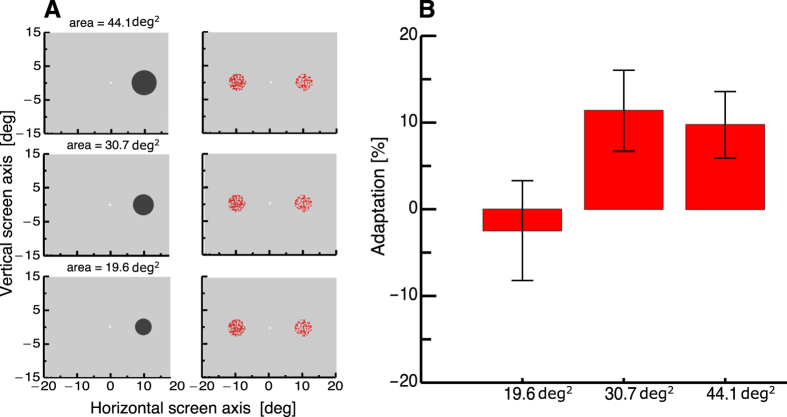
(**A**) Experimental set-up for testing numerosity judgments after adaptation to visual size. The procedure was identical to the main experiment, except that only a single adapter was shown on the right side of the screen. The size of the adapter disk varied to be either as big as the probe patch, or bigger by a factor of 1.5 or 2.25. After adaptation, two numerosity patches were shown. The probe patch (on the right side of the screen) always contained 100 dots. (**B**) Average biases in numerosity judgments after adaptation to the three adapter disks. Error bars represent SEM.

We ran a separate control experiment in order to check whether the adaptation of probe patches presented after the large adapter resulted from local contrast adaptation. In that case dots might have been missed or made indiscernible from each other. We presented three adaptation conditions in which the adapter either had the same size as the probe patch or two bigger sizes. Size adaptation was only expected when the size adapter was bigger than the probe patch. As can be seen in Fig. 4, no numerosity adaptation occurred in the condition where the adapter was as big as the probe patch. A one-way repeated measures ANOVA confirmed significantly stronger adaptation in the two condition where the adapter was bigger than the probe patch (F(2,12) = 4.75, p = 0.034).

## Discussion

The main finding of this study is that low numerosities are invariant to changes in apparent patch size whereas the perception of high numerosities is affected by adaptation to visual size. The data suggest that the contribution of patch area to the calculation of dot numerosity depends logarithmically on how many dots are presented in the patch.

Changing the size of an image, for example with image processing software, would entail a shift in the density of objects but not their numerosity. With our size adaptation method, however, we observed the opposite in visual perception: Density remained constant, but numerosity estimation changed. This result can be explained if size and density are processed independently, whereas numerosity processing is modulated by information about stimulus size. Evidence from brain imaging suggest that density information is processed in areas V4 and TEO^16^, while the estimation of stimulus size occurs in area V1^11^. Numerosity detection, however, involves higher areas in the intraparietal cortex^3^,^4^. The estimation of stimulus density can be driven in two ways: First, by averaging the local distances between the dots or second by counting the dots and relating the numerosity to the size of the area on which the dots are displayed. The latter mechanism would involve that density processing is informed about stimulus size and therefore should be affected by adaptation. However, no effect of size adaptation on density perception was found in the current study.

From a neuroanatomic perspective, numerosity processing has access to size and density information and therefore could combine the two types of information to construct an estimate of numerosity. In that case, numerosity perception should be strongly affected by adaptive changes in stimulus size. We found that this is true for high numerosities (>25 dots). This is consistent with earlier studies, which found differences between low and high numerosity processing. Anobile *et al*.^14^ for instance showed that low numerosities obey Weber’s law, whereas higher numerosities do not. These data depended on the density of the dots: For low densities thresholds increased directly with numerosity whereas for higher densities, thresholds increased with the square root of density. We did not find a dependence of our adaptation effects on physical dot density. The logarithmic relationship between numerosity and size adaptation was virtually identical when tested with small (19.6 deg^2^) and large (154 deg^2^) number clouds. The reason for this difference may be either because the size adaptation method adds variance to the data, thus making it not sensitive enough to measure Weber’s law. Please remember, that we adapted probe and reference stimulus simultaneously. Another reason might be the dot size which in our study was half the size of those used by Anobile *et al*.^14^. Smaller dots might be more discernible and thereby less affected by density perception mechanisms. Additionally, perception of low numerosities is invariant to changes in area size^13^ but perception of high numerosities is not^8^. This evidence suggests that low dot numerosities are sensed directly via neurons that are tuned for specific numerosities^7^,^17^. The numerosities of higher dot numerosities, however, are inferred on the basis of visual cues as size and density. Such effects of visual cues have previously been reported: Dakin *et al*.^8^ tested perception of 128 dots and found that manipulations of area size affected numerosity and density judgments. Gebuis and Reyvoet^11^,^12^ tested smaller numerosities (12–44 dots) while systematically varying parameters such as convex hull, average diameter, aggregate surface, and density. They found that visual cues had an influence on numerosity judgments. It must be stressed that in our experiment size adaptation did also slightly affect judgments of low numerosities. However, the effects of size adaptation on higher numerosities were much stronger. The most parsimonious explanation for this effect is that the divide between low and high numerosities is not binary but gradual. In this view, the influence of visual cues rises with increasing dot numerosities.

Imaging studies indicate that the cerebral representations of size and numerosity overlap^18^,^3^. Recently, Harvey *et al*.^3^ reported a large overlap of maps coding the perceived numerosity and size in human intraparietal cortex. However, whereas object size tuning was best described by linear functions, tuning for numerosity was characterized by logarithmic functions (but see the comment by Gebuis *et al*.)^19^. Numerosities represented in the intraparietal sulcus are strongly influenced by topological invariants, such as connectivity and the inside/outside relationship^20^ The overlapping representations for size and numerosity are likely to constitute the neural basis for the here observed adaptation effects. Especially, the resemblance of those neural tuning properties to our data, where numerosity perception depended logarithmically on size adaptation, supports this hypothesis.

In conclusion, we found that adaptation to size distorts numerosity perception of subsequently shown numerosity patches. Adaptation of numerosity perception increased logarithmically with the presented numerosity of dots in the patches. Density perception remained unaffected by adaptation to size.

## Materials and Methods

### Participants

Six subjects (5 female, one male, mean age 32 years) participated in main experiment with small numerosity clouds. Five subjects (4 female, one male, mean age 25 years) took part In the experiment involving large numerosity clouds. In the control experiment seven different subjects (3 female, 4 male, mean age 29 years) participated. All had normal or corrected to normal vision and were naive to the purpose of the experiment. Experiments were carried out in accordance with the Declaration of Helsinki. Participants were remunerated for their time. All experiments of the study were approved by the ethics committee of the German Society of Psychology (DGPS) and conducted in accordance with their guideline.

### Apparatus

Subjects were seated 57 cm from a Samsung Sync-Master 2233 (Seoul, South Korea) with their head stabilized by a chin- and headrest. The visible screen diagonal was 20 inches, resulting in a visual field of 40 deg × 30 deg. Stimuli were presented on the monitor with a vertical frequency of 60 Hz on a homogeneously gray background (41.8 cd/m^2^).

### Procedure

Each trial consisted of an adaptation phase and a test phase. Subjects had to maintain their gaze on a fixation point, presented at the center of the screen, throughout the whole experiment. In the adaptation phase, two disks (one with a small area size of 1.13 deg^2^ and one with a large area size of 30.68 deg^2^) were shown for 5000 ms, centered at the horizontal meridian and at a horizontal eccentricity of 7.8 deg, i.e., to the left and the right of the fixation point (see Fig. 1A). The adapter disks changed contrast polarity with 0.5 Hz. After offset of the adapter disks two random dot patches (dot radius: 0.15 deg, dot color: red) were presented for 600 ms, each with an area size of 19.6 deg^2^, centered at the same position as the adapters.

The probe patch contained either 4, 7, 9, 13, 17, 21, 25, 50, 75, or 100 dots. For each of the 10 dot numerosities in the probe patch, the threshold of apparent numerosity was measured with the method of constant stimuli. For this reason the reference patch, which had the same size as the probe patch, contained for each of the dot numerosities in the probe patch one out of 7 equiprobable (−90% to +90% of the probe patch numerosity) dot numerosities. Subjects were instructed to report which patch contained more numerosities by pressing the left or right arrow key. Subjects were asked to report which patch contained the higher density, In separate sessions the apparent size of the probe patch was measured for all 10 dot numerosities. In these sessions the size of the probe patch on the right side was held constant (area: 19.6 deg^2^) and the size of the reference patch on the left side was varied (from 1 deg to 4 deg, in 7 equiprobable steps). Subjects were instructed to report which patch was larger by pressing the left or right arrow key. The order of numerosity presentation within each session and the order of session were randomized. When subjects pressed one of the arrow keys the next trial started immediately. Numerosity, density and size judgements were each measured in separate sessions. For each of the three judgments, seventy trials were measured for each of the ten numerosity thresholds.

In separate sessions, we tested the same experiment with the only exception that number clouds spread over an area size of 154 deg^2^. The size of the adapters in this control sessions were 240.5 deg^2^ and 9.6 deg^2^.

### Control Experiment

In a separate Control Experiment, we tested how the size of the large adapter influenced numerosity perception. The adapter disk (with an alternating contrast polarity of 0.5 Hz) was shown with one of three area sizes: 19.6, 30.7, or 44.1 deg^2^. After offset of the adapter disk, two numerosity patches were shown at a horizontal eccentricity of 7.8 deg, i.e., to the left and the right of the fixation point. The probe patch on the right was presented in each trial with a radius of 2.5 deg containing 100 dots. The comparison patch had the same size, but the numerosity of dots it contained varied across trials, as in the main Experiment. A full psychometric function was measured for each of the three adapter sizes. The order of conditions within each session was randomised across trials. For each of the three conditions, seventy trials were measured for each of the ten numerosity thresholds.

### Ethical statement

Written informed consent was obtained prior to each experiment in accordance with the Declaration of Helsinki. Participants were remunerated for their time. All experiments of the study were approved by the ethics committee of the German Society of Psychology (DGPS) and conducted in accordance with their guideline.

## Additional Information

**How to cite this article**: Zimmermann, E. and Fink, G. R. Numerosity perception after size adaptation. *Sci. Rep.* **6**, 32810; doi: 10.1038/srep32810 (2016).
